# Urea cycle disorders in India: clinical course, biochemical and genetic investigations, and prenatal testing

**DOI:** 10.1186/s13023-018-0908-1

**Published:** 2018-10-01

**Authors:** Sunita Bijarnia-Mahay, Johannes Häberle, Anil B. Jalan, Ratna Dua Puri, Sudha Kohli, Ketki Kudalkar, Véronique Rüfenacht, Deepti Gupta, Deepshikha Maurya, Jyotsna Verma, Yosuke Shigematsu, Seiji Yamaguchi, Renu Saxena, Ishwar C. Verma

**Affiliations:** 1 Institute of Medical Genetics and Genomics, Sir Ganga Ram Hospital, New Delhi, India; 2 University Children’s Hospital Zurich and Children’s Research Centre, Steinwiesstr 75, CH-8032 Zurich, Switzerland; 3 Navi Mumbai Institute of Research In Mental And Neurological Handicap (NIRMAN), Navi Mumbai, India; 4 Department of Pediatrics, Faculty of Medical Science, University of Fukui, Fukui, Japan; 5 Department of Pediatrics, Shimane University Faculty of Medicine, 89-1 En-ya-cho Izumo, Shimane, 693-8501 Japan

**Keywords:** Urea cycle, UCD, OTC deficiency, Citrullinemia, Argininosuccinic aciduria, Mutation, Prenatal diagnosis, Hyperammonemia

## Abstract

**Background:**

Urea cycle disorders (UCDs) are inherited metabolic disorders that present with hyperammonemia, and cause significant mortality and morbidity in infants and children. These disorders are not well reported in the Indian population, due to lack of a thorough study of the clinical and molecular profile.

**Results:**

We present data from two major metabolic centres in India, including 123 cases of various UCDs. The majority of them (72/123, 58%) presented in the neonatal period (before 30 days of age) with 88% on or before day 7 of life (classical presentation), and had a high mortality (64/72, 88%). Citrullinemia type 1 was the most common UCD, observed in 61/123 patients. Ornithine transcarbamylase (OTC) deficiency was the next most common, seen in 24 cases. Argininosuccinic aciduria was diagnosed in 20 cases. Deficiencies of arginase, N-acetylglutamate synthase, carbamoyl phosphate synthetase, citrin, and lysinuric protein intolerance were also observed.

Molecular genetic analysis revealed two common *ASS1* mutations: c.470G > A (p.Arg157His) and c.1168G > A (p.Gly390Arg) (36 of 55 tested patients). In addition, few recurrent point mutations in *ASL* gene, and a deletion of the whole *OTC* gene were also noted. A total of 24 novel mutations were observed in the various genes studied. We observed a poor clinical outcome with an overall all time mortality of 63% (70/110 cases with a known follow-up), and disability in 70% (28/40) among the survivors. Prenatal diagnosis was performed in 30 pregnancies in 25 families, including one pre-implantation genetic diagnosis.

**Conclusions:**

We report the occurrence of UCDs in India and the spectrum that may be different from the rest of the world. Citrullinemia type 1 was the most common UCD observed in the cohort. Increasing awareness amongst clinicians will improve outcomes through early diagnosis and timely treatment. Genetic diagnosis in the proband will enable prenatal/pre-implantation diagnosis in subsequent pregnancies.

**Electronic supplementary material:**

The online version of this article (10.1186/s13023-018-0908-1) contains supplementary material, which is available to authorized users.

## Background

Urea Cycle Disorders (UCD) are a group of inborn errors of metabolism seen frequently in tertiary care intensive care units (ICUs) in view of the hyperammonemic encephalopathy which is the most common presenting feature. The overall incidence of UCD varies from 1:8000 to 1:44,000 births [1–4]. A more recent study from 3 countries in Europe reported an estimated cumulative incidence of 1 in 51,946 for UCDs [5]. Incidence of UCD is not known in India. All types of UCDs have been recognized.

The overall outcome of children with UCD remains poor, even in the developed world where excellent infrastructure and facilities are available [1, 6]. Children with UCD are recognized after symptoms of hyperammonemia such as lethargy and coma occur, thus leading to a significant delay in diagnosis. This may happen in babies who are screened as well, because neonatal hyperammonemic encephalopathy often occur before neonatal screening test results are known. A delay in diagnosis results in either death or neurodisability [7, 8]. This is exacerbated by a lack of awareness amongst families and primary health care physicians, causing a delay in seeking medical attention. Additional factors in developing countries like India are inadequate facilities in most primary and secondary level hospitals that contribute to a poor outcome. For instance, laboratory ammonia assays are usually only available even in tertiary hospitals. Therefore, a majority of babies die without a diagnosis, or are recognized very late, contributing to increased morbidity and mortality. An accurate genetic diagnosis is essential not only for appropriate management of the child, but also for genetic counseling in the context of subsequent pregnancies and carrier testing. So far, only a few mutations have been reported from India [9–12]. The Indian population is a unique mix of several cultures and sects, with considerable consanguinity. With the distinctness of Indian gene pool, mutations may be different from other populations and perhaps recurrent in some areas in view of closed community marriages, limiting the gene pool to an ethnic group.

We present here our experience in diagnosis, genetic testing and outcome after diagnosis of UCD in these families. The data was collected from two major metabolic centres in India.

## Methods

### Patient cohort

All neonates and children presenting with hyperammonemia and biochemical/clinical features of UCD since year 2001 till December 2017 were included in the cohort. The patients were collected from two centres. In the first centre at Sir Ganga Ram Hospital, New Delhi, they were chosen either from the genetic clinic or from the molecular genetics laboratory, which received samples for genetic testing for UCD from referring clinicians. A biochemical diagnosis of UCD was taken as the inclusion criteria. In some cases, where DNA of the index patient was not available, parents of the deceased patients were enrolled in the study, where good clinical and biochemical data was available to fulfill the diagnostic criteria. This centre was designated as Centre 1. In addition, cases from another referral centre (Centre 2) in Mumbai (NIRMAN) were also included in this study. All cases with complete clinical, biochemical and molecular genetic data were taken. All parents or legal guardians provided their consent for DNA testing. All the tests were carried out as part of clinical care as diagnostically indicated.

For selection of cases, information of clinical symptoms and hyperammonemia were solicited. This was followed by biochemical analysis of amino acids by Tandem mass spectrometry (MS/MS) on dried blood spots (DBS) or Ultra/ High performance liquid chromatography (U/HPLC) on plasma samples, and quantification of urinary orotic acid by Gas chromatography-mass spectrometry (GC-MS). To confirm the diagnosis of UCD, genetic testing was carried out on DNA extracted from peripheral blood or dried blood spots from affected children or their parents, where the index case’s sample was not available. Sequencing of the genes was performed either at Münster University in Germany (2001–2008) or University Children’s Hospital in Zurich (2008–2017) or at Sir Ganga Ram Hospital (2008–2017) in New Delhi. Genetic testing was done in cases from both centres. Prenatal diagnosis using chorionic villous sampling (CVS) or amniocentesis and mutation analysis was performed in subsequent pregnancies of families.

### Molecular studies for UCD gene mutations

DNA was isolated from human samples using standard methods. PCR products were purified using Multiscreen^R^ PCR_μ96_ plate (MilliPore, USA). Bidirectional sequencing of the coding exons and corresponding intron/exon boundaries were set up using the same primers as for PCR, with purified PCR products using BigDye® Terminator v 3.1 cycle sequencing kit (Applied Biosystems, California). Sequencing was carried out on ABI 3500Dx automated sequencer unit (Applied Biosystems). Sequence chromatograms obtained were analyzed with Chromas software (Technelysium, Tewantin, Australia) and by manual inspection.

Obtained sequences were compared by blasting them against the reference genomic sequences (for *ASS1*: NM_000050, NP000041; for *ASL*: NM_000048, NP_000039; for *OTC*: NM_000531, NP_000522). Bioinformatics analysis was carried out to determine the functional consequences of mutations and their effects on protein structure by using different tools, e.g., Polyphen2 (genetics.bwh.harvard.edu/pph2/), Mutation Taster (www.mutationtaster.org/), or Sorting Intolerant from Tolerant (http://provean.jcvi.org/index.php) [13–15].

The probands with suspected OTC deficiency or their mothers (in case of non-availability of DNA from proband due to death prior to diagnosis), were evaluated for deletions/duplications in the *OTC* gene by (Multiplex ligation probe amplification) MLPA probe mix (MRC Holland P079-OTC). Mutation analyses at Münster or Zurich University were done as reported [16–19].

For one patient, cDNA analysis of *CPS1* gene was performed. RNA-isolation and cDNA-synthesis was performed using an established protocol [20]. Amplification by PCR and direct sequencing of the carbamoylphosphate synthetase 1 transcript was done in 6 overlapping fragments. Ref. sequences: GenBank NM_001875.4 and Ensembl ENSG00000021826, ENST00000233072.

## Results

A total of 148 patients from the two centres were collected, of whom 123 patients from 123 families fulfilled the criteria and were included in the study. Nine patients from Centre 1, and 16 cases from Centre 2 were excluded because of lack of a confirmed diagnosis. Seventy seven cases from Centre 1 and 48 from Centre 2 fulfilled the criteria and were enrolled. In the cohort of patients whose data was collected, 82% have been after year 2007.

The diagnosis was established in all 123 patients; 61 had citrullinemia type 1, 26 patients had OTC deficiency, 20 were of argininosuccinic aciduria (ASA), 9 had arginase deficiency, 3 had carbamoyl phosphate synthetase (CPS1), 2 had N-acetyl glutamate synthase (NAGS) deficiency and one patient each had lysinuric protein intolerance (LPI) and citrin deficiency (Table 1). All patients were diagnosed biochemically by quantitative amino acid analysis on dried blood spots (MS/MS) or in plasma (U/HPLC), except for LPI where both plasma and urine amino acid quantitation was performed by HPLC (see Additional file 1).

**Table 1:** Clinical features and outcomes of the various urea cycle disorders

Types of UCD	ASS1-D	ASL-D	OTC-D	ARG-D	CPS1-D	NAGS-D	Citrin-D	LPI	Total
Total Numbers	61	20	26	9	3	2	1	1	123
Consanguinity	9 (*n* = 54)	7 (*n* = 19)	2 (*n* = 25)	5 (*n* = 9)	1 (n = 2)	1 (n = 2)	0 (n = 1)	1 (n = 1)	26 (*n* = 113)
Positive family history	14 (*n* = 56)	9 (*n* = 20)	12 (*n* = 24)	2 (n = 9)	0 (*n* = 3)	1 (n = 1)	0 (n = 1)	0 (n = 1)	38 (*n* = 115)
Age group									
Neonatal	44	13	11	0	2	2	0	0	72
Infancy	6	3	4	0	0	0	0	0	13
> Infancy (> 1 year)	11	4	11	9	1	0	1	1	38
Clinical presentation									
Neonatal or intermittent encephalopathy, or seizures	51	17	19	0	3	1	0	1	92
Liver disease (deranged liver enzymes)	6	5	2	0	0	0	0	0	13
Failure to thrive	3	1	1	1	0	0	0	1	7
Developmental delay	6	5	7	9	0	0	0	0	27
Other neuropsychiatric/ behavioural/ ataxia/ spasticity symptoms	Nil	0	4	6	1	0	1	0	12
Mutations performed	55	16	22	5	3	2	1	1	105
Results obtained	55	15	18	5	3	2	1	1	100
Outcome									
Death	44	12	11	0	1	2	0	0	70
Alive	10	5	14	8	1	0	1	1	40
Development: Delayed	7	5	7	8	1	0	0	0	28
Development: Normal or near normal	2	1	7	0	0	0	1	1	12
Diet -Protein restriction+ medications	6	5	10	8	1	0	0	1	31
Lost to follow up/ not known	7	3	1	1	1	0	0	0	13

*ASS1-D* Argininosuccinate synthetase type 1 deficiency, *ASL-D* Argininosucccinate lyase deficiency, *OTC-D* Ornithine transcarbamylase deficiency, *ARG-D* Arginase deficiency, *CPS1-D* CPS1 deficiency, *NAGS-D* N-acetyl glutamate synthase deficiency, *Citrin-D* Citrin deficiency, *LPI* Lysinuric protein intolerance

Clinical details were available for all 123 patients. Full clinical, laboratory, and outcome details are available in Additional file 1. Approximately three-fifth of patients (72/123, 58%) presented in the first 30 days of life (neonatal period), 88% of whom presented on or before day 7 of life (classical presentation). Thirteen cases presented in infancy between 1 and 12 months of life, and 38 after the first year of life. Overall male to female ratio was 1.7:1 (2.25:1 in OTC deficiency, 1.65:1 in non-OTC deficiency). Consanguinity and a positive family history were noted in 23% (26/113) and 33% (38/115) of families, respectively. All families were unrelated except one where a non-consanguineous couple visited our clinic after losing their daughter on day 6 of life with encephalopathy and no other investigations. Husband’s niece was detected to have citrullinemia type 1. Based on the history, the couple was tested and detected to be carriers of citrullinemia type 1. Apart from this, there were no related families.

Detailed description of all cases including demography, clinical presentations, family history, biochemical investigations, outcomes and prenatal diagnosis data (for subsequent pregnancies in parents of probands) are shown in Additional file 1: Table S1. A large number of cases presented with encephalopathy with or without seizures (94/123, 76%), either in neonatal period or later (Table 1). Half of the patients in the late onset group presented with developmental delay (22/51, 43%). A significant number of children had features of liver disease (10.5%) and failure to thrive (3%) (Table 1).

### Outcome analysis

**Fig. 1:**
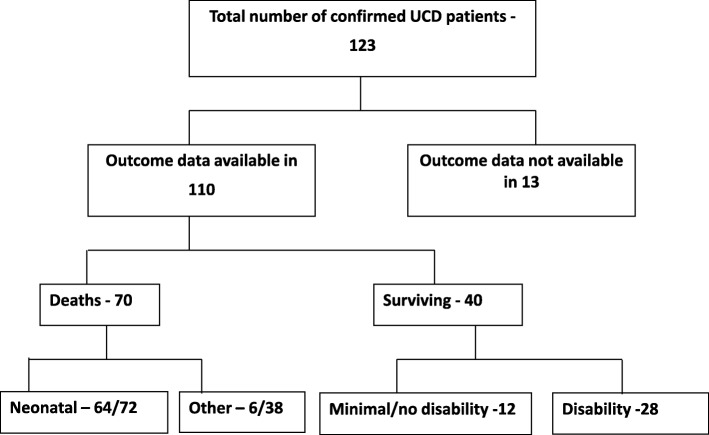
Flowchart showing outcomes of UCD patients

Outcome data was available from 110 cases. Of these, 70 died and 12 patients are healthy and developing normally. The remaining 28 patients are surviving with varying degree of disability (Fig. 1 and Table 1).

Citrullinemia type 1 was the most frequently observed UCD and showed the worst outcome. Of 55 patients for whom outcome was known, 44 died (81.5%), 7 children survived with disability ranging from minor learning difficulties to severe global delay and seizures, and 3 children survived without disability: 2 of whom received liver transplantation, and 1 patient with a milder presentation of neonatal cholestasis and liver dysfunction that resolved over time. All except 2 deaths occurred in the first episode of illness in neonatal period. In the two children who survived the initial insult in neonatal period, there was severe failure to thrive, and death because of relentless progression of disease with intercurrent illnesses.

Outcomes in ASL and OTC deficiency cases were also poor with a total mortality of 50% (23/46 cases). In ASL deficiency survivors, all except 1 had some degree of disability. In OTC deficiency survivors, 5 of 8 girls and 2 of 6 boys have developmental delay and 3 girls and 4 boys were healthy (Table 1, Additional file 1: Table S1). All the children who are doing well presented with mild symptoms of episodic vomiting.

### Molecular genetic studies

Molecular studies could be performed in 105 patients/families. All tests were targeted sequencing of genes based on preliminary biochemical diagnosis. Pathogenic or likely pathogenic variants were noted in 100 cases. Molecular analysis for specific genes did not reveal any pathogenic variant in 5 cases. Details of mutations are listed in Tables 2, 3 and 4. Table 5 provides details of clinical presentation, and gene studies performed in mutation negative patients.

**Table 2:** Details of mutations in all patients

Gene	Mutation				Number of cases		No. of alleles	Reference	ExAC data base (allele frequency)
	Position	DNA	Protein/ mRNA	Type	Homo zygous	Hetero/ Hemi zygous			
ASS1	Exon 5	c.190G > A	p.Val64Ile	Missense	1	1	3	Novel	0.00003303
	Exon 5	c.269G > A	p.Gly90Asp	Missense	1	0	2	Novel	NA
	Exon 5	c.271A > C	p.Thr91Pro	Missense	1	0	2	Novel	0.00003324
	Exon 5	c.299G > A	p.Arg100His	Missense	0	1	1	[30]	0.0001597
	Exon 5	c.349G > T	p.Gly117Cys	Missense	1	0	2	[30]	NA
	Exon 5	c.350G > A	p.Gly117Asp	Missense	0	1	1	[36]	NA
	Exon 6	c.370G > A	p.Asp124Asn	Missense	1	1	3	[37]	NA
	Exon 7	c.470G > A	p.Arg157His	Missense	12	1	25	[38]	0.00009932
	Exon 9	c.570T > A	p.Tyr190Ter	PTC**	0	1	1	Novel	NA
	Exon 12	c.793C > T	p.Arg265Cys	Missense	3	1	7	[39]	0.000008262
	Exon 12	c.815G > A	p.Arg272His	Missense	2	0	4	[40]	NA
	Exon 13	c.910C > T	p.Arg304Trp	Missense	2	0	4	[38]	0.00004119
	Exon 14	c.970G > A	p.Gly324Ser	Missense	1	0	2	[38]	0.00004952
	Exon 14	c.1088G > A	p.Arg363Gln	Missense	0	1	1	[30]	0.000008792
	Exon 15	c.1139delA	p.Glu380Argfs*20	Deletion sbp*	2	1	5	Novel	NA
	Exon 15	c.1168G > C	p.Gly390Arg	Missense	23	1	47	[38]	NA
	Total				50	10	110		
ASL	Exon 3	c.89_94delinsGTCGTA	p.Tyr30_Asp31delinsCysArg	DEL INS***	0	1	1	Novel	NA
	Exon 5	c.326C > G	p.Thr109Arg	Missense	0	1	1	Novel	NA
	Exon 5	c.337C > T	p.Arg113Trp	Missense	1	0	2	[16]	0.00006783
	Exon 7	c.509G > A	p.Ser170Asn	Missense	0	2	2	[41]	NA
	Exon 8	c.593C > T	p.Pro198Leu	Missense	1	0	2	Novel	NA
	Exon 9	c.637C > T	p.Arg213Ter	PTC	0	3	3	[42]	0.000008417
	Exon 9	c.649C > T	p.Arg217Ter	PTC	1	0	2	Novel	0.000008443
	Exon 11	c.733T > C	p.Trp245Arg	Missense	0	1	1	Novel	NA
	Exon 11	c.749T > A	p.Met250Lys	Missense	0	1	1	Novel	0.00000854
	Exon 12	c.857A > G	p.Gln286Arg	Missense	2	1	5	[43]	0.00007499
	Exon 12	c.913G > A	p.Gly305Arg	Missense	0	1	1	Novel	NA
	Exon 12	c.967A > G	p.Lys323Glu	Missense	1	0	2	Novel	NA
	Exon 12	c.978G > C	p.Gln326His	Missense	0	1	1	[41]	0.00003771
	Exon 16	c.1153C > T	p.Arg385Cys	Missense	1	0	2	[44]	0.00006732
	Exon 17	c.1297A > C	p.Ser433Arg	Missense	0	2	2	[41]	NA
	Exon 17	c.1300G > T	p.Val434Leu	Missense	0	2	2	[41]	0.000008284
	Total				7	16	30		
OTC	Exon 1	c.274C > T	p.Arg92Ter	PTC	0	1	1	[45]	NA
	Exon 1	c.275G > A	p.Arg92Gln	Missense	0	1	1	[45]	NA
	Exon 4	c.386G > A	p.Arg129His	Missense	0	3	3	[32]	NA
	Exon 5	c.421C > T	p.Arg141Ter	PTC	0	1	1	[33]	NA
	Exon 5	c.535C > T	p.Leu179Phe	Missense	0	1	1	[46]	NA
	Exon 6	c.604C > T	p.His202Tyr	Missense	0	1	1	[47]	NA
	Exon 7	c.674C > T	p.Pro225Leu	Missense	0	2	2	[48]	NA
	Exon 7–8	c.773_790del	p.Asn258_263del	Deletion 18 bp	0	1	1	Novel	NA
	Exon 8	c.805G > A	p.Gly269Arg	Missense	0	1	1	Novel	NA
	Exon 8	c.829C > T	p.Arg277Trp	Missense	0	1	1	[49]	NA
	Exon 8	c.835C > T	p.Gln279Ter	PTC	0	1	1	[50]	NA
	Exon1–10	Whole gene deletion		Large deletion	0	4	4	[51]	NA
	Total				0	18	18		
ARG1	Exon 1	c.2 T > C	p.Met1?	Initiation codon	1	0	2	Novel	NA
	Exon 3	c.132_146del	p.Gln44_Lys48del	Deletion 15 bp	0	1	1	Novel	NA
	Exon 3	c.295G > A	p.Gly99Arg	Missense	0	1	1	Novel	0.00001658
	Exon 5	c.551delC	p.Pro184Leufs*7	PTC	1	0	2	Novel	NA
	Intron 7	c.802 + 2 T > G	Splice site	Splice site	1	0	2	Novel	NA
	Exon 8	c.877delG	p.Val293Ter	PTC	1	0	2	Novel	NA
	Total				4	2	10		
CPS1	Intron 3	c.236 + 4A > G (NM_001875.4)	Splice site	Splice site	1	0	2	Novel	
	Exon 19	c.2339G > A	p.Arg780His	Missense	1	0	2	[52]	0.00003304
	Exon 21	c.2623A > G	p.Lys875Glu	Missense	1	0	2	[18]	0.002427
NAGS	Exon 3	c.787G > T	p.Glu263Ter	PTC	1	0	2	Novel	NA
	Exon 4	c.991C > T	p.Gln331Ter	PTC	1	0	2	Novel	NA
SLC25A13	Exon 7	c.650delT	p.Phe217Serfs*33	Deletion sbp	0	1	1	[11]	
	Exon 9	c.869T > C	p.Ile290Thr	Missense	0	1	1	[11]	0.000008256
SLC7A7	Exon 15	c.158C > T	p.Ser53Leu	Missense	1	0	2	[12]	NA
GRAND TOTAL					67	48	100		

*sbp single base pair, **PTC Premature termination codon, ***DEL INS Deletion insertion

**Table 3:** Evaluation of pathogenicity of novel variants using in-silico prediction tools

Gene	Mutation	Protein change	Mutation taster	LRT	SIFT	PROVEAN	DANN	ExAC	dbSNP
ASS1	c.190G > A	p.Val64Ile	Disease causing	Neutral	Tolerated	Neutral	0.9791	0.00003303	556,297,791
	c.269G > A	p.Gly90Asp	Disease causing	Unknown	Damaging	Damaging	0.9985	Not Present	1,422,867,920
	c.271A > C	p.Thr91Pro	Disease causing	Unknown	Damaging	Damaging	0.9963	0.00003324	769,018,733
	c.570C > A	p.Tyr190Ter	Disease causing	Unknown	NA	NA	0.9957	Not Present	Not Present
	c.1139delA	p.Gln380Argfs*20	Disease causing	NA	NA	NA	NA	Not Present	1,213,378,896
ASL	c.89_94delinsGTCGTA	p.Tyr30_Asp31delinsCysArg	Disease causing	NA	NA	NA	NA	Not present	Not present
	c.326C > G	p.Thr109Arg	Disease causing	Deleterious	Damaging	Damaging	0.9938	Not present	Not Present
	c.593C > T	p.Pro198Leu	Disease causing	Deleterious	Damaging	Damaging	0.9992	Not Present	1,282,829,485
	c.649C > T	p.Arg217Ter	Disease causing	Neutral	NA	NA	0.9972	0.000008443	369,879,957
	c.733T > C	p.Trp245Arg	Disease causing	Deleterious	Damaging	Damaging	0.9941	Not present	Not Present
	c.749T > A	p.Met250Lys	Disease causing	Deleterious	Damaging	Damaging	0.9791	Not present	754,634,171
	c.913G > A	p.Gly305Arg	Disease causing	Deleterious	Damaging	Damaging	0.9993	Not present	Not Present
	c.967A > G	p.Lys323Glu	Disease causing	Deleterious	Damaging	Damaging	0.9987	Not present	Not Present
OTC	c.773_790del	p.Asn258_263Del	Disease causing	NA	NA	NA	NA	Not present	Not Present
	c.805G > A	p.Gly269Arg	Disease causing	Deleterious	Damaging	Damaging	0.9992	Not Present	Not Present
ARG1	c.2T > C	p.Met1Thr	Disease causing	Deleterious	Damaging	Neutral	0.9809	Not Present	Not Present
	c.132_146del	p.Gln44_Lys48del	Disease causing	NA	NA	NA	NA	Not Present	Not Present
	c.295G > A	p.Gly99Arg	Disease causing	Deleterious	Damaging	Damaging	0.9993	0.00001658	753,829,097
	c.551delC	p.Pro184Leufs*7	Disease causing	NA	NA	NA	NA	Not Present	Not Present
	c.802 + 2 T > G	Splice site	Disease causing	NA	NA	NA	0.9948	Not Present	Not Present
	c.877delG	p.Val293Ter	Disease causing	NA	NA	NA	NA	Not Present	Not Present
CPS1	c.254 + 4A > G	Splice site	Disease causing	NA	NA	NA	0.9747	Not Present	Not Present
NAGS	c.787G > T	p.Glu263Ter	Disease causing	Deleterious	NA	NA	0.9963	Not Present	Not Present
	c.991C > T	p.Gln331Ter	Disease causing	Deleterious	NA	NA	0.9973	Not Present	1,445,639,047

Mutation Taster: An in silico prediction tool for the pathogenicity of a variant based on evolutionary conservation, splice-site, mRNA, protein and regulatory features. The potential is predicted by a naive Bayes classifier

LRT: Likelihood ratio test (LRT) predicts deleterious variants through identification of highly conserved amino acid regions using a comparative genomics data set of 32 vertebrate species. Range 0 to 1

SIFT: SIFT (sorts intolerant from tolerant) is an in silico prediction tool for nonsynonymous variants based on sequence homology derived from closely related sequences collected through PSI-BLAST. Range 0 to 1 with values less than 0.05 usually considered intolerant. 40% of the values in this database are below 0.01

PROVEAN: Protein Variation Effect Analyzer is an in silico tool that predicts how nonsynonymous, MNP, or in-frame indel variant will affect a protein’s biological function. The prediction is based on alignment-based scores derived from pairwise sequence alignments between the query sequence and each of the related sequences at the protein level. Range − 14 to + 14

DANN: DANN is a pathogenicity scoring methodology developed by Daniel Quang, Yifei Chen and Xiaohui Xie at the University of California, Irvine. It is based on deep neural networks. The value range is 0 to 1, with 1 given to the variants predicted to be the most damaging

**Table 4:** Types of mutations detected

Total number of patients with mutations	100
Number of patients with homozygous mutations	67
Total number of mutations	58
Total number of novel mutations	24
Missense mutations	39
Premature termination codon	10
Initiation codon mutation	1
Splice site mutation	2
Small insertions/ deletions	5
Large deletion	1

**Table 5:** Patients in whom molecular studies showed no mutations

Gene studied	S.No (as in Additional file 1)	Age at presentation	Sex (proband)	Consanguinity	Family history of similar disease	Clinical features in proband	Amino acids levels by MS/MS on dried blood spot (μmol/l)	GC-MS in urine	Ammonia level (in μmol/l)
ASL	78	4 days	Male	No	No	Neonatal encephalopathy, convulsions, lethargy, reduced feeding	Citrulline- 2557, ASA- 753.19	Increased orotate 393	NK
OTC	86	4 days	Male	No	Yes, previous 2	Neonatal encephalopathy and deaths	NK	Allopurinol challenge test in mother - increase in orotate	NK
	93	5 days	Male	No	Yes	Neonatal encephalopathy and death	NK	Increased orotate	High
	96	2 years	Female	No	Yes, elder sister	Encephalopathy, Febrile illness with high ammonia	Normal amino acid levels Citrulline 15, arginine 15, ornithine 146	Increased orotate	High 1600
	98	1.5 years	Female	No	No	Encephalopathy, Vomiting, awkward behaviour, high ammonia	NK	Increased orotate	Level NK

A total of 58 different mutations were detected in 100 patients, 16 of which were observed in *ASS1* gene and *ASL* gene each, 12 mutations in *OTC* gene, and 6 mutations each in *ARG1* gene, 3 in *CPS1,* 2 in *NAGS* and *SLC25A13* genes, and one in *SLC7A7* gene (Table 2). Twenty four mutations were novel. Of 100 patients in whom the diagnosis was genetically confirmed, 67 were homozygous and 15 were compound heterozygous, 12 were hemizygous and 6 were heterozygous (for an *OTC* mutation).

*ASS1* gene sequencing was performed in 55 out of 61 cases. The two most common mutations, c.470G > A (p.Arg157His) and c.1168G > A (p.Gly390Arg) were observed in 72 alleles out of a total of 110 alleles (65.5%). The missense mutation, c.1168G > A occurred twice more frequently (47/110 alleles [42.7%]) than c.470G > A (25/110 alleles [22.7%]). Five mutations were novel (Table 3).

Sequencing of *ASL* gene revealed more varied findings with 16 mutations in 15 cases. Interestingly, all mutations were in compound heterozygous states in the 8 cases from Centre 1 whereas all mutations were noted in homozygous form in the patients from Centre 2. Four mutations were noted to be recurrent: c.509G > A (p.Ser170Asn), c.637C > T (p.Arg213Ter), c.1297A > C (p.Ser433Arg) and c.1300G > T (p.Val434Leu), occurring in 2, 3, 2 and 2 alleles, respectively. Eight mutations were noted to be novel.

*OTC* gene sequencing was performed in 22 of 26 cases. No mutation was identified in 4 cases (one male, 2 female probands and 1 carrier mother) after full sequencing of *OTC* gene as well as MLPA analysis for deletions/duplications. The most common mutation noted was a whole gene deletion in 4 cases, accounting for 22.2% (4/18) cases. Rest of mutations were single nucleotide substitutions, missense or nonsense mutations. Pathogenic variants were also identified in arginase deficiency (5 cases), CPS1 deficiency (3 cases), two cases of NAGS deficiency, one case each citrin deficiency [10] and LPI [11]. All pathogenic variants in *ARG1* and *NAGS* genes were novel (Tables 2 and 3). Another novel pathogenic variant was noted in *CPS1* gene. The pathogenicity of this variant, c.236 + 4A > G (NM_001875.4) was checked via RNA studies at Zurich. On RNA level, as a consequence of this splice-site mutation, two alternatively spliced transcript variants were observed: the majority of transcripts showed complete skipping of exon 2 (r.127_236del, p.Ala43Valfs*7) but also partial skipping of exon 2 (r.206_236del, p.Glu70Thrfs*70) was observed. Both parents are carriers of the mutation, confirming obligate heterozygosity.

### Prenatal diagnosis

Mutation analysis led to successful prenatal diagnoses in 30 pregnancies in 25 families (Table 6). This included one couple that opted for pre-implantation genetic diagnosis (PGD) after having an affected fetus in a preceding pregnancy. In this case the result was normal, and the couple delivered a healthy baby. Of the 29 prenatal analyses, 11 pregnancies were found to carry fetuses with no mutation (normal), 13 with carrier fetuses (including 2 OTC female carriers, both of whom are doing well after birth) and 5 carried affected fetuses. Amongst 19 pregnancies in families with ASS or ASL deficiency, 4 pregnancies were found to have fetuses affected with disease (21%).

**Table 6:** Prenatal diagnosis in families with UCDs

S. No (as per Additional file 1)	Diagnosis in proband	Age at presentation in probands	Consanguinity among parents	Mutations in proband	Protein change	Number of PND	Results of PND
5	ASS1-D	6 days	No	c.1168G > A	p.Gly390Arg	Once	Not affected (carrier)
7	ASS1-D	5 days	Yes	c.470G > A	p.Arg157His	Twice	One affected, one unaffected (carrier)
10	ASS1-D	5 days	No	c.1168G > A	p.Gly390Arg	Once	Not affected
16	ASS1-D	7 days	No	c.570T > A c.350G > A	p.Tyr190Ter & p.Gly117Asp	Once	Affected
17	ASS1-D	4 days	No	c.1139delA	p.Glu380Arg fs*20	Once	Not affected (carrier)
18	ASS1-D	4 days	No	c.470G > A	p.Arg157His	Once, one PGD	Affected, PGD pregnancy healthy baby
19	ASS1-D	6 days	Yes	c.970G > A	p.Gly324Ser	Once	Not affected (carrier)
22	ASS1-D	2 days	Yes	c.470G > A	p.Arg157His	Once	Not affected (carrier)
27	ASS1-D	4 days	No	c.1168G > A	p.Gly390Arg	Once	Not affected
30	ASS1-D	5 days	No	c.470G > A	p.Arg157His	Once	Not affected (carrier)
33	ASS1-D	3 days	No	c.1168G > A	p.Gly390Arg	Once	Not affected
35	ASS1-D	Neonatal	No	c.1168G > A	p.Gly390Arg	Once	Not affected
61	ASS1-D	Neonatal	No	c.1168G > A	p.Gly390Arg	Once	Not affected (carrier)
62	ASL-D	4 days	No	c.509G > A c.1297A > C	p.Ser170Asn p.Ser433Arg	Twice	Affected once, not affected second time
65	ASL-D	5 days	No	c.637C > T c.1300G > T	p.Arg213Ter p.Val434Leu	Once	Not affected (carrier)
70	ASL-D	9 days	Yes	c.913G > A c.749 T > A	p.Gly305Arg p.Met250Lys	Once	Not affected (carrier)
87	OTC-D	6 days	No	c.674C > T	p.Pro225Leu	Twice	Not affected (once carrier, once no mutation)
91	OTC-D	3 days	Yes	whole gene deletion (mother tested)		Once	Carrier female
92	OTC-D	3 days	No	c.274C > T	p.Arg92Ter	Once	Not affected
94	OTC-D	5 days	Yes	c.421C > T	p.Arg141Ter	Twice	First affected male, second carrier female
97	OTC-D	2 days	No	whole gene deletion (mother tested)		Once	Not affected
101	OTC-D	5 years	No	whole gene deletion (mother tested)		Once	Not affected
119	CPS1 -D	NK	NK	c.2623A > G	p.Lys875Glu	Once	Not affected
120	NAGS-D	NK	NK	c.991C > T	p.Gln331Ter	Once	Not affected
121	NAGS-D	Neonatal	Yes	c.787G > T	p.Glu263Ter	Once	Not affected (Carrier)

NK: Not known, PGD: Pre-implantation genetic diagnosis

## Discussion

Urea cycle disorders occur universally with varying frequencies in different populations [1, 21–23]. While OTC deficiency has been reported to be the most commonly occurring UCD, citrullinemia type 1 is reported to be less common [3, 5, 24]. In contrast, in our cohort, citrullinemia type 1 contributed to about half (61/123, 49.6%), thus making citrullinemia type 1 the most commonly occurring UCD in this cohort. The data was pooled from two centres, both showing similar results independently. OTC deficiency was observed with lower frequency in the cohort. In fact, the total incidence for OTC deficiency was less than half of that found for citrullinemia type 1. Although suspected in few additional patients (hyperammonemia with high urinary orotic acid expected in OTC deficiency), there was insufficient evidence to prove OTC deficiency in some patients because of the difficulty in performing enzyme based diagnosis (which would have required a liver biopsy) or due to lack of mutations in the *OTC* gene despite full gene study (sequencing as well as testing for larger deletions and duplications by MLPA). These difficulties are however well known and described earlier in large studies [25]. Many factors contributed to the failure to achieve a definitive diagnosis in all patients: the lack of availability of enzyme diagnostics at any centre in India, the invasiveness of the test (liver biopsy) along with difficulty in shipment of liver biopsy specimens in frozen state to another centre abroad, and the overall cost involved. It is therefore likely that cases of OTC deficiency are being missed in India, as known from literature [25].

The situation is different in the case of citrullinemia type 1, where a very high citrulline level on dried blood spot MS/MS can be reliably detected leading to further gene studies confirming the diagnosis. Presence of ASA in blood or urine is indicative of ASL deficiency, and for elevated plasma arginine of arginase deficiency.

Urea cycle disorder commoner than OTC deficiency has also been noted in Saudi Arabian population, where ASL deficiency has been described as the most common UCD. Consanguinity in this particular population seems to be one leading explanation [26]. Parallels can be drawn with the Indian population where both consanguinity and closed community marriages are the norm, leading to genetic isolates. This may be a part explanation to citrullinemia type 1 being more common in India. In our cohort, consanguinity was noted in 23% families. Consanguinity and close community marriages are important factors leading to increased occurrence of recessive disorders, at least in India [27].

In our study, 10 couples (and 6 expectant mothers in case of OTC deficiency) were tested in absence of a sample from index patient, and their gene study detected heterozygous mutations, suggesting an accurate diagnosis in their probands. In seven couples, citrullinemia type 1 was suspected based on elevated plasma citrulline in index patient and these couples were only tested for mutations in *ASS1* gene.

The presentation in most Indian children was similar to what is known in literature, with both neonatal classic form and late onset presentations. In our cohort, the neonatal classic presentation was more common in all UCDs except arginase and citrin deficiency. This finding is somewhat different from other studies where majority of presentations are beyond first 30 days of life [1, 28, 29]. The more common early presentation in our cohort may have been a bias because neonatal cases are recognized more easily in view of a careful medical supervision in the neonatal period. A recent study from Europe reported 50 patients of UCD detected both symptomatically (39/50) and NBS/presymptomatically (11/50). In this cohort, 28 of 39 symptomatically detected patients presented with neonatal encephalopathy, thus corroborating with our observation of a neonatal presentation being more common [5]. The high mortality among the neonatal encephalopathy group in our cohort was similar to the cohort reported from Europe [5].

A family history of previous sibling deaths was present in approximately 33% of families. This highlights the immense burden of the disease on the families, and the ignorance as well as lack of facilities denying these families of an opportunity for early diagnosis and prompt treatment. This would have prevented the suffering, either by successful treatment of the index child or by prenatal diagnosis. Molecular diagnosis thus helped the families in prevention of recurrence of this difficult group of disorders.

Molecular analysis revealed several interesting findings besides being immensely helpful in family counseling. For *ASS1* gene, there were two common mutations, p.Gly390Arg and p.Arg157His which constituted 65.5% of cases. These two are previously reported mutations and are present globally [30]. Most common mutation seen in multiple ethnic group is p.Gly390Arg except in the east Asian population where another mutation, c.421-2A > G is the commonest [31]. Another mutation, p.Arg265Cys seen in our population was common to the Japanese population [31]. There seemed to be a good genotype-phenotype correlation, which is in agreement with reported literature [24]. Two recurring mutations, p.Arg213Ter and p.Ser170Asn, were found in *ASL* gene.

In a significant number of OTC deficiency patients, a whole gene deletion was detected. In this disorder, whole gene deletions as well as nonsense mutations were noted either in neonatally presenting severely affected males or in manifesting females, while missense mutations were noted in mildly affected boys. This finding is also in agreement with literature [21, 32, 33]. The mutation p.Arg129His, showing a mild phenotype with excellent prognosis, has previously been described [34]. Similarly, p.Arg277Trp is known to be a mild mutation, reported even in male transmission [35].

Molecular analysis enabled successful prenatal diagnosis in 30 pregnancies with no false negative results. This is an effective way of reducing the disease-related morbidity and mortality, until appropriate facilities are made available for early diagnosis and treatment.

## Conclusions

This is the first comprehensive report of mutations in UCDs from the Indian subcontinent. Increasing availability of gene sequencing has enabled higher numbers of prenatal diagnosis for UCDs. Establishing a precise molecular diagnosis by mutation analysis is helpful not only for accurate genetic counseling but also for accurate carrier testing, which is an efficient way for reducing the morbidity and mortality associated with the disease through prenatal diagnosis.

## Additional file

Additional file 1: Details of clinical, biochemical and molecular analysis and of outcomes and prenatal diagnoses in each patient in the study. The data provides complete information about each patient enrolled in the study. This includes the demographic details, clinical and family history, the laboratory investigations (biochemical as well as mutation analysis), an outcome data and information about prenatal diagnosis in the particular family. Each patient is assigned a number, and all patients belong to different families, thus 123 patients from 123 families were enrolled. Data is pooled from two genetic centres differentiated by use of different color font in the table – black for centre 1 and maroon for centre 2. (XLSX 30 kb)

## Abbreviations

ARG: Arginase
ASL: Argininosuccinate Lyase
ASS1: Argininosuccinate Synthetase 1
CPS1: Carbamoyl phosphate synthetase 1
GCMS: Gas chromatography mass spectrometry
HPLC: High performance liquid chromatography
ICU: Intensive Care Units
MS/MS: Mass spectrometry, mass spectrometry/ Tandem mass spectrometry
NAGS: N-acetyl glutamate synthase
OTC: Ornithine Transcarbamylase
UCD: Urea cycle disorders
